# Ferrous ascorbate as a potential biomarker for diabetic retinopathy: a vitreous humour metabolomics study

**DOI:** 10.1186/s12886-024-03530-6

**Published:** 2024-06-24

**Authors:** Jinmeng Liu, Shuang Liu, Peng Hui, Siying Teng, Jinghui Xie, Yabin Sun

**Affiliations:** Ophthalmology Department, First Hospital of Jilin University, No. 1 Xinmin Street, Changchun, 130021 Jilin province China

**Keywords:** Diabetic retinopathy, Vitreous humour, Metabolomics, Ferrous ascorbate, Biomarkers

## Abstract

**Background:**

This study aimed to explore differences in vitreous humour metabolites and metabolic pathways between patients with and without diabetic retinopathy (DR) and identify potential metabolite biomarkers.

**Methods:**

Clinical data and vitreous fluid samples were collected from 125 patients (40 without diabetes, 85 with DR). The metabolite profiles of the vitreous fluid samples were analysed using ultra-high performance liquid chromatography, Q-Exactive mass spectrometry, and multivariate statistical analysis. A machine learning model based on Least Absolute Shrinkage and Selection Operator Regularized logistic regression was used to build a risk scoring model based on selected metabolite levels. Candidate metabolites were regressed to glycated haemoglobin levels by a logistic regression model.

**Results:**

Twenty differential metabolites were identified between the DR and control groups and were significantly enriched in five Kyoto Encyclopedia of Genes and Genomes pathways (arginine biosynthesis; tricarboxylic acid cycle; alanine, aspartate, and glutamate metabolism; tyrosine metabolism; and D-glutamate metabolism). Ferrous ascorbate significantly contributes to poorer glycaemic control outcomes, offering insights into potential new pathogenic pathways in DR.

**Conclusions:**

Disorders in the metabolic pathways of arginine biosynthesis, tricarboxylic acid cycle, alanine, aspartate, glutamate metabolism, tyrosine metabolism, and D-glutamate metabolism were associated with DR. Risk scores based on vitreous fluid metabolites can be used for the diagnosis and management of DR. Ferrous ascorbate can provide insights into potential new pathogenic pathways for DR.

**Supplementary Information:**

The online version contains supplementary material available at 10.1186/s12886-024-03530-6.

## Background

Diabetic retinopathy (DR) is the most common microvascular complication in patients with diabetes and the leading cause of visual impairment and blindness worldwide. The International Diabetes Federation projects that the global population with diabetes will reach 700 million by 2045 [1]. Patients with long-term diabetes develop macrovascular complications, including heart disease, stroke, and peripheral arterial obstructive disease which eventually lead to death in 70% of cases; microvascular complications mainly cause nephropathy, retinopathy, and neuropathy [2]. Among the risk factors for DR progression, haemoglobin A1c (HbAlc) has the greatest impact. Compared with patients with HbAlc < 7.0%, those with HbAlc > 10.0% have a higher risk of progression to incident DR, referable DR, diabetic macular oedema, and proliferative diabetic retinopathy (PDR) [3]. The United Kingdom Prospective Diabetes Study reported that prolonged hyperglycaemia exposure leads to negative metabolic memory that reduces the potential impact of good glycaemic control, highlighting the necessity for early and appropriate treatment of hyperglycaemia and associated metabolic disorders [4].

Untargeted metabolomic analysis is an unbiased investigation of all metabolites within a sample and can reveal biologically relevant changes within a system. Previous metabolomic studies in patients with pre-diabetes and type 2 diabetes revealed that changes in amino acid and lipid concentrations can be used as biomarkers to identify at-risk patients and monitor disease progression and treatment efficacy [5]. Metabolic and functional changes of retinal tissue, along with systemic reactions during DR progression, can lead to structural and molecular alterations in the vitreous, reflecting pathological events at the vitreoretinal interface and characterising the diabetic condition. Consequently, vitreous alterations can impact the diabetic retina pathologically, contributing to a vicious cycle of disease progression [6]. Because the vitreous is attached to the retina, structural and biochemical changes in the vitreous can reflect the pathophysiological processes in retinal tissue [7]. Therefore, vitreous fluid-based metabolomic studies contribute to the understanding of DR pathogenesis and development of new therapeutic targets.

To date, the incomplete metabolic profile of the vitreous in healthy and diseased states has hindered differentiation of metabolic characteristics in diseased retinas [8], resulting in limited descriptions of human vitreous metabolism focused on a small subset of metabolites. Barba et al. observed increased lactate and decreased ascorbate in a metabolomic analysis of vitreous humor from 22 patients with PDR and 22 non-diabetic patients with macular lentigines [9]. Paris et al. examined vitreous humour samples from 20 PDR patients and 31 non-diabetic patients with anterior retinal or macular tears, reporting increased levels of arginine, proline, and allantoin [10]. Additionally, Haines et al. noted an increase in pyruvate and purine-related pathways [8]. While these initial studies lay the groundwork for future research, they are insufficient to construct a complete metabolic profile and require expanded sample sizes for thorough investigation. Therefore, we included vitreous humour from patients with diabetic retinopathy and retinopathy without diabetes in our study to identify metabolic signatures of the disease through untargeted metabolomics.

This non-targeted metabolomics study of vitreous fluid obtained from patients with DR used liquid chromatography coupled with high-resolution mass spectrometry (LC-MS) with the aim to investigate changes in metabolites and metabolic pathways in the vitreous fluid of patients with DR, which can help identify new therapeutic targets.

## Methods

### Study participants and sample collection

**Fig. 1:**
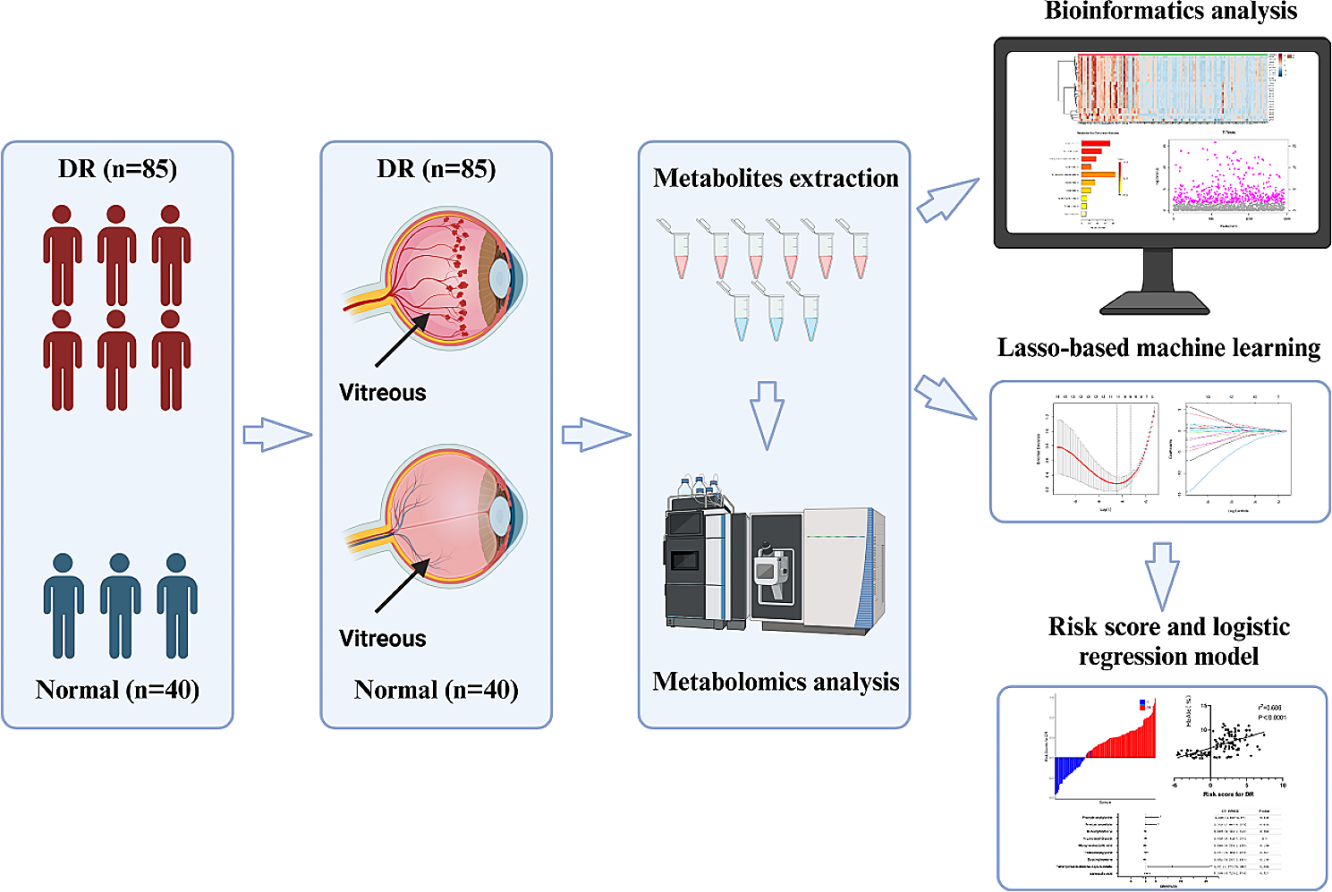
Initial study of human vitreous metabolism

The study was conducted at the Ophthalmology Department of First Hospital of Jilin University between January 2022 and March 2023. The protocol was approved by the Ethics Committee of the First Hospital of Jilin University and conducted in accordance with the ethical standards for human experimentation and Declaration of Helsinki (2013). Participants signed informed consent before vitreous fluid samples were collected. Inclusion criteria for the experimental group comprised type 2 diabetic patients with DR requiring vitrectomy, while the control group consisted of non-diabetic patients with macular lentigines, retinal lentigines, retinal detachments, and macular antrums necessitating vitrectomy. Clinical data and vitreous fluid samples were collected from 125 patients (Fig. 1). Exclusion criteria for both groups included [11]: (1) having other ocular diseases (including glaucoma and uveitis); (2) history of ocular surgery; (3) undergoing anti-VEGF therapy; and (4) history of severe systemic inflammatory disease. The experimental group included 85 patients with diabetes mellitus type 2 with DR. The control group included 40 patients without diabetes with macular fissure (*n* = 7), retinal fissure (*n* = 1), retinal detachment (*n* = 26), and pre-macular membrane (*n* = 6). DR was classified into non-proliferative DR (NPDR) and PDR based on the presence or absence of neovascularisation. The early stage of proliferation was characterised by the presence of neovascularisation of the retina elsewhere or neovascularisation of the disc. The fibro-proliferative stage was characterised by the presence of a fibrovascular membrane, which may be combined with preretinal haemorrhage or vitreous haemorrhage. The late proliferative stage was characterised by the presence of retinal detachment, which may be combined with fibrovascular membrane, preretinal haemorrhage, or vitreous haemorrhage. All patients underwent a preoperative ophthalmologic examination. The 85 patients in the experimental group were categorised into NPDR (*n* = 8), early proliferative stage (*n* = 7), fibroproliferative stage (*n* = 23), or late proliferative stage (*n* = 47).

Descriptive statistics for demographic and clinical variables were calculated. Medical history, age, sex body mass index (BMI), and duration of disease were obtained. Blood and urine laboratory tests included fasting blood glucose (FBG), glycosylated HbA1c, serum creatinine (SCR), triglycerides (TG), high-density lipoprotein cholesterol (HDL-C), low-density lipoprotein cholesterol (LDL-C), total cholesterol (TC), retinol-binding protein (RBP), recombinant apolipoprotein A1, and apolipoprotein B. Data were analysed using IBM SPSS Statistics for Windows, version 27.0 (IBMCorp., Armonk, N.Y., USA). Missing data were removed sequentially. Nonparametric data were expressed as median (interquartile spacing), parametric data as mean ± SD, and categorical variables as numbers and percentages. Differences between categorical variables were assessed using the chi-square and Fisher’s exact tests. Independent samples t-tests were used to calculate significant differences between parametric continuous variables, and the Wilcoxon rank-sum test was applied to nonparametric continuous variables. Statistical significance was set at *p* < 0.05.

The procedures were performed by the same experienced chief surgeon at the First Hospital of the Jilin University Ophthalmology Department. Vitreous sampling was performed by the same ophthalmologist to prevent systematic errors. Vitreous fluid samples were collected during vitrectomy, 0.3 mL of vitreous was collected and stored in sterile tubes, which were immediately cooled and stored at -80 °C [11].

### Sample preparation for metabolomics

Overall, 50 µL of vitreous fluid was added to 100 µL of methanol: acetonitrile (1:l, V/V) [12]. The mixed sample was vortexed for 30 s at 4 °C and sonicated for 10 min (ice bath). The sonicated sample was incubated in a refrigerator at -20 °C for 1 h and centrifuged at 4 °C and at high speed (15,000 rpm) for 15 min. The supernatant was collected, placed in an injection vial, and injected into the sample for analysis. Biological quality control (QC) samples were made from 10 µL of each sample from all groups, and QC samples were inserted into the sample cohort to monitor and assess system stability.

### Liquid chromatography/mass spectrometry

The metabolites were separated using an ACQUITY UPLC system (Waters, USA) [13, 14]. The liquid phase conditions were performed as specified (see Table S1, Additional file 1, which demonstrates liquid phase conditions).

Ion detection was performed using a Q-Exactive quadrupole electrostatic field orbital trap ultrahigh-resolution mass spectrometer. Quantification was performed by simultaneous scanning of positive and negative ions. Mass spectrometry conditions and acquisition methods were shown in Tables S2 and S3, Additional file 1.

### Statistical analyses

The raw data were processed for peak detection, extraction, alignment, and integration using Compound Discoverer software. The pre-processed raw data were imported into Metaboanalyst 5.0 (Xia Lab, McGill University, Montreal, Canada) for multivariate statistical analysis after normalisation, data transformation, and scaling for principal component analysis (PCA) and orthogonal partial least squares discriminant analysis (OPLS-DA) using a two-sample t-test to identify altered metabolites in patients with univariate-level DR [15, 16]. Metabolites with variable importance of prediction threshold (VIP) values > 1 in the OPLS-DA analysis and *p* < 0.05 in the univariate analysis were considered significantly different metabolites and receiver operating characteristic (ROC) curves were plotted. The obtained differential metabolite kernel ratios and ion patterns were imported into the Human Metabolome Database (HMDB) to identify metabolites [17, 18] and correlation analysis was performed with R package ‘corrplot’. Pathway enrichment analysis was performed by Metaboanalyst 5.0 to screen out the differential metabolic pathways that could significantly differentiate (*p* < 0.05) between DR patients and healthy controls. The Least Absolute Shrinkage and Selection Operator Regularized logistic regression (LASSO-LR) feature selection method was performed by the R package ‘glmnet’ to evaluate the discriminatory ability of each feature for the sample categories, obtain the distribution of the content of the filtered features in the two groups, construct the model by taking the Ln of the quantitative values of the metabolites, and obtain the scoring formula of the risk score [19]. Pearson’s linear correlation analysis between DR risk score and HbAlc was performed using SPSS. To further explore the association between vitreous humour metabolites and systemic glycaemic control, HbAlc > 6.5% was set as the ending variable, and the raw data of the nine metabolites were set as independent variables after normalisation by sum, log transformation, and auto-scaling, respectively, and were measured by age, sex, presence of hypertension, BMI, systolic blood pressure (SBP), diastolic blood pressure (DBP), TG, TC, HDL-c, LDL-c, FBG, serum creatinine, RBP, and duration of diabetes mellitus were used as cofactors to adjust the logistic regression model. The results are presented by plotting the odds ratios (ORs) values, 95% confidence intervals (CIs), and *p*-values on a forest plot using GraphPad Prism 9.5.0 for Windows (GraphPad Software, San Diego, California USA).

## Results

### Clinical features of patients

Table 1 presents the demographic and clinical information of vitreous samples from the study population. The results indicated a significantly higher prevalence of hypertension in the DR experimental group compared to the control group (*p* = 0.001). Additionally, levels of SBP (*p* = 0.03), HbA1c (*p* < 0.001), FBG (*p* < 0.001), SCR (*p* = 0.006), and RBP (*p* = 0.001) were significantly higher than in the control group. In terms of complications, patients in the DR experimental group were significantly more prone to nephropathy, neuropathy, and heart disease than those in the control group. Metabolites were affected by the lens and partially originated from circulating plasma before filtration by the ciliary body. No significant difference in IOL ocular status was observed between the experimental and control groups, indicating that lens status did not influence the study results.

**Table 1:** Demographic and clinical information of the study population

Variables	Control (*n* = 40)	DR (*n* = 85)	*p*-value
Sex (male/female)*	12/28	37/48	0.148
Age (years)†	60 (52,65)	55 (51,60)	0.068
Pseudophakia (yes/no)%*	1/39	4/81	0.922
Intraocular surgeries (yes/no)%*	1/39	4/81	0.922
SBP (mmHg)‡	136.12 ± 19.9	145 ± 21.68	0.03^*^
DBP (mmHg)‡	85.33 ± 11.08	84.13 ± 13.57	0.628
Hypertension (yes/no)%*	12/28	51/34	0.001^**^
BMI (kg/m^2^)‡	24.95 ± 4.17	25.58 ± 3.15	0.401
HbA1c (%)†	4.8 (4.57,5.15)	7.9 (6.6,9)	< 0.001^**^
FBG (mmol/L)†	5.41 (4.95,6)	7.3 (5.15,9.67)	< 0.001^**^
SCR (µmol/L)†	60.7 (51.73,74.65)	78.4 (56.2,114.5)	0.006
TC (mmol/L)‡	4.69 ± 1.09	5.09 ± 1.32	0.1
HLD-c (mmol/L)†	1.11 (0.98,1.18)	1.13 (0.98,1.28)	0.404
LDL-c (mmol/L)†	3.08 (2.54,3.62)	3.11 (2.67,3.98)	0.388
TG (mmol/L)†	1.9 (1.08,2.81)	1.54 (0.99,2.78)	0.537
RBP (mg/L)†	41.65 (38.4,53.9)	57 (45,73)	0.001^**^
Apoa-1 (g/L)‡	1.3 ± 0.18	1.25 ± 0.23	0.434
ApoB‡	0.96 ± 0.2	0.94 ± 0.26	0.76
Duration of DM (years)	-	10(5,17)	-
Complications			
Nephropathy (yes/no) %	1/39(2.5%)	12/73 (14.12%)	0.095
Neuropathy (yes/no) %	0/40(0%)	13/72 (15.29%)	0.022^*^
Heart disease (yes/no) %	3/37(7.5%)	10/75 (11.76%)	0.678
DME (yes/no) %	-	6/79 (7.06%)	-
Treatment			
Insulin treatment (yes/no) %	-	79/6 (92.94%)	-
Metformin treatment (yes/no)%	-	15/70 (17.65%)	-
Photocoagulation (yes/no) %	-	31/54 (36.47%)	-

Non-parametric data are presented as median (interquartile range) and parametric data as mean ± SD. *p < 0.05 and **p < 0.01

* Chi-square test

†Mann–Whitney–Wilcoxon test

‡Independent samples t-test

SBP, systolic blood pressure; DBP, diastolic blood pressure; BMI, body mass index; FBG, fasting blood glucose; TC, total cholesterol; HDL-C, high-density lipoprotein; LDL-C, low-density lipoprotein; TG, triglycerides; RBP, Retinol-Binding Protein; DME, diabetic macular oedema

**Table 2:** Differential metabolic markers identified in metabolomics analysis

Metabolite	Fold change (E/C)	VIP	Elements	m/z	Reference Ion	Trend	AUC	*p*-value
Phenylalanylglycine	7.50	1.05	HMDB0028995	223.11	[M + H] + 1	↑	0.91	3.04E-04
Tetrahydroaldosterone-3-glucuronide	2.29	1.25	HMDB0010357	539.25	[M-H]-1	↑	0.89	9.37E-05
Cortolone-3-glucuronide	2.82	1.16	HMDB0010320	541.27	[M-H]-1	↑	0.87	1.52E-04
6-[3-(2-carboxyethyl)-5-hydroxyphenoxy]-3,4, 5-trihydroxyoxane-2-carboxylic acid	3.45	1.11	HMDB0128026	359.10	[M + H] + 1	↑	0.86	1.20E-04
L-Glutamic acid 5-phosphate	3.01	1.03	HMDB0001228	228.03	[M + H] + 1	↑	0.86	2.31E-04
Ferrous ascorbate	3.49	1.02	HMDB0303656	232.97	[M + H] + 1	↑	0.84	8.51E-04
S-Nitrosoglutathione	2.41	1.49	HMDB0004645	335.07	[M-H]-1	↑	0.82	2.17E-06
Succinylacetone	0.21	2.12	HMDB0000635	159.07	[M + H] + 1	↓	0.77	1.99E-14
N-Acetylhistidine	0.29	2.18	HMDB0032055	198.09	[M + H] + 1	↓	0.72	1.31E-16
Uridine 5’-diphosphate	0.25	1.65	HMDB0000295	405.01	[M + H] + 1	↓	0.65	3.63E-09
Maleylacetoacetic acid	0.36	1.81	HMDB0002052	199.03	[M-H]-1	↓	0.61	7.92E-13
Malonic acid	0.38	1.98	HMDB0000691	103.00	[M-H]-1	↓	0.60	7.35E-15
N-Linoleoyl Glycine	0.30	1.74	HMDB0241917	270.26	[M + H] + 1	↓	0.59	5.32E-13
Fumaric acid	0.39	1.34	HMDB0000134	115.00	[M-H]-1	↓	0.57	1.19E-07
Oxoglutaric acid	0.41	1.13	HMDB0000208	145.01	[M-H]-1	↓	0.56	1.08E-05
Selenocystine	0.47	1.64	HMDB0004122	334.91	[M-H]-1	↓	0.56	1.46E-09
Threonic acid	0.41	1.82	HMDB0000943	135.03	[M-H]-1	↓	0.55	2.37E-11
N-palmitoyl asparagine	0.36	1.11	HMDB0241921	261.04	[M + H] + 1	↓	0.55	1.62E-06
Thioxanthine monophosphate	0.43	1.76	HMDB0060876	264.98	[M + H] + 1	↓	0.53	2.12E-11
Tridecanoylglycine	0.29	1.44	HMDB0013317	272.22	[M + H] + 1	↓	0.52	1.13E-06

The trend indicates that the expression level of the differential metabolite in the experimental group shows either an increase or decrease compared to the control group. “↑” indicates an increase and “↓” indicates a decrease

### Analysis of metabolic profiles and metabolic pathways

**Fig. 2:**
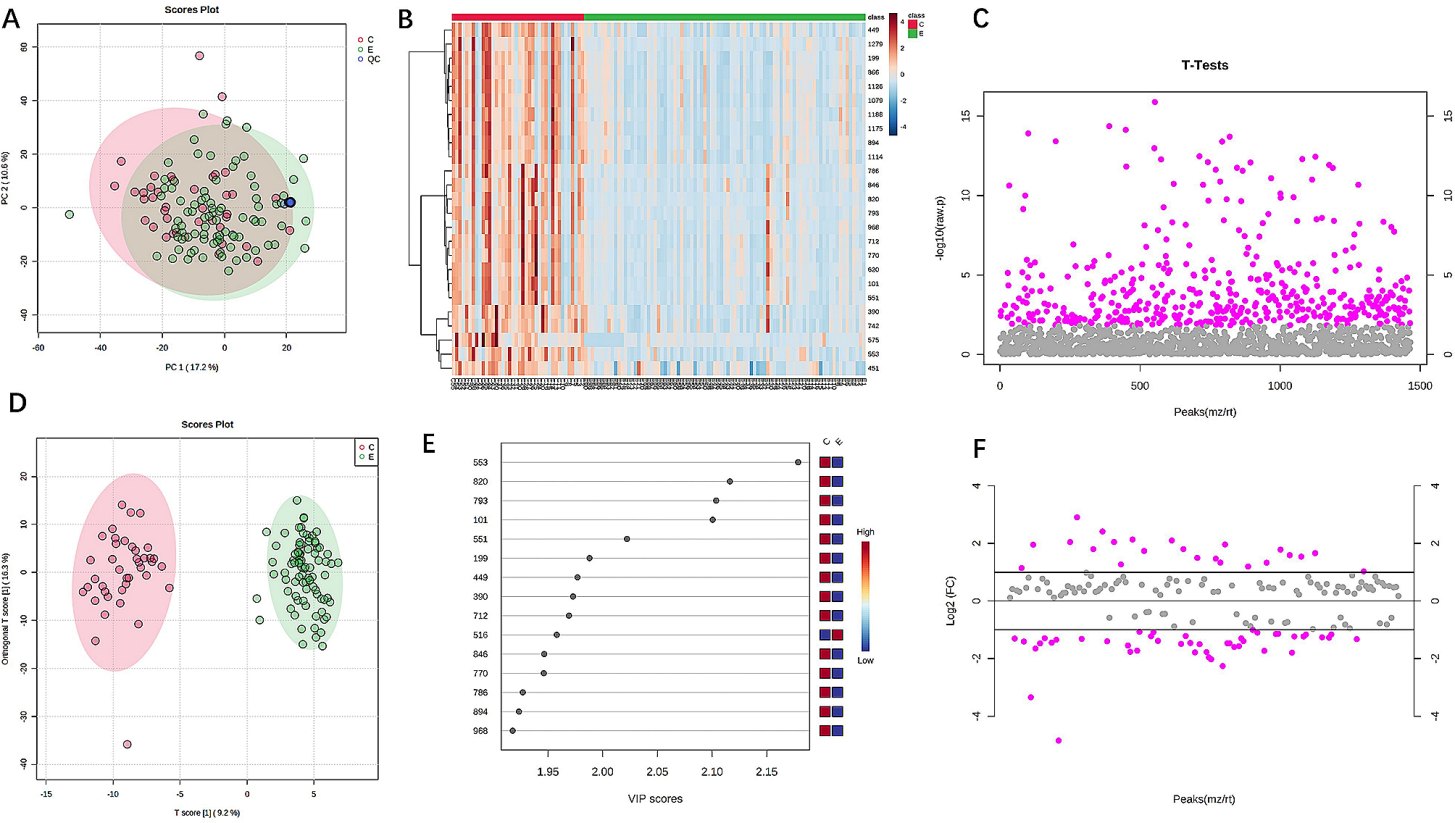
(**A**) Two-dimensional score map of the PCA model: red indicates the control group, green the experimental group, and blue the QC group, with good reproducibility within and between-group variability. (**B**) Heatmap of hierarchical clustering: each coloured cell on the map corresponds to a concentration value in the data table, identifying clusters with increasing (orange) or decreasing (blue) metabolite profiles. (**C**) Two-sample t-test: Metabolites screened for differences at *P* < 0.05 are in red, and non-significant metabolites in grey. (**D**) OPLS-DA score plot: red indicates the control group, green indicates the experimental group, the plot indicates a large difference between groups and within groups. (**E**) VIP-spot: the horizontal coordinate is the VIP value, and the vertical coordinate is the metabolite marker. (**F**) FC analysis: red indicates differential metabolites exceeding a given threshold (FC > 2 or FC < 0.5), and grey indicates non-significant metabolites. FC, fold change; OPLS-DA, orthogonal partial least squares discriminant analysis; PCA, principal component analysis

**Fig. 3:**
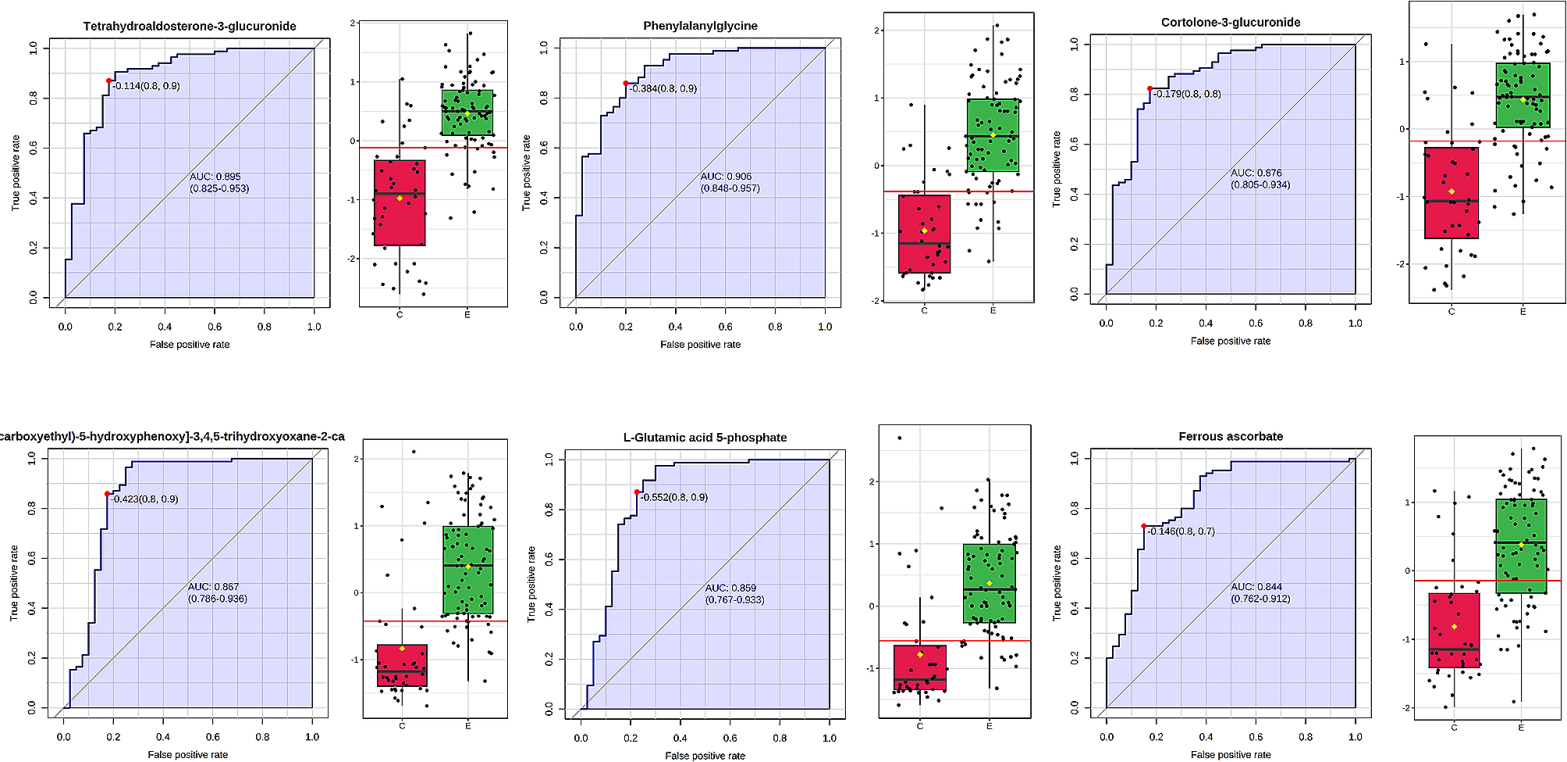
ROC curves: Y-axis: sensitivity, X-axis: 1-specificity (false positive rate). ROC, receiver operating characteristic

A total of 1,465 metabolites were identified in vitreous fluid samples collected from 85 patients in the DR experimental group and 40 non-diabetic control patients, respectively. The experimental and control groups showed significant separation and differences in both PCA (Fig. 2A) and thermograms (Fig. 2B), indicating good differences between groups. A total of 456 metabolites with *p* < 0.05 were screened using a two-sample t-test (Fig. 2C). OPLS-DA (Fig. 2D) was performed to obtain 169 metabolites associated with DR according to the VIP value of (Fig. 2E). A total of 69 metabolites with fold change (FC) > 2 or FC < 0.5 were screened by FC analysis (Fig. 2F), with 23 metabolites showing upregulated expression levels and 46 metabolites showing decreased expression levels in the experimental group. The mass-to-core ratios and ion patterns corresponding to the 69 screened metabolites were imported into the HMDB, and 20 differential metabolites were identified (Table 2). The 11 differential metabolites in positive ion mode were N-acetylhistidine, N-linoleoyl glycine, thioxanthine monophosphate, tridecanoylglycine, uridine 5’-diphosphate, succinylacetone, N-palmitoyl asparagine, phenylalanylglycine, ferrous ascorbate, 6-[3-(2-carboxyethyl)-5-hydroxyphenoxy]-3,4,5-trihydroxyoxane-2-carboxylic acid, and L-glutamic acid 5-phosphate. The nine differential metabolites in negative ion mode were threonic acid, oxoglutaric acid, fumaric acid, malonic acid, S-nitrosoglutathione, maleylacetoacetic acid, cortolone-3-glucuronide, tetrahydroaldosterone-3-glucuronide, and selenocystine. The sensitivity of the identified metabolites was assessed by ROC curve analysis (see Figure S1, Additional file 1, which shows ROC curves for 20 differential metabolites). The following top six metabolites were selected as DR candidate biomarkers: tetrahydroaldosterone-3-glucuronide, phenylalanylglycine, cortolone-3-glucuronide, 6-[3-(2-carboxyethyl)-5-hydroxyphenoxy]-3,4,5-trihydroxyoxane-2-carboxylic acid, L-glutamic acid 5-phosphate, and ferrous ascorbate, with *p*-values of 0.00030374,0.0000937, 0.00015204, 0.00012039, 0.00023063, and 0.00085142, respectively, and areas under the curve (AUCs) of 0.895 (95% CI = 0.825–0.953), 0.906 (95% CI = 0.848–0.957), 0.876 (95% CI = 0.805–0.934), 0.867 (95% CI = 0.786–0.936), 0.859 (95% CI = 0.767–0.933), and 0.844 (95% CI = 0.762–0.912), respectively (Fig. 3).

**Fig. 4:**
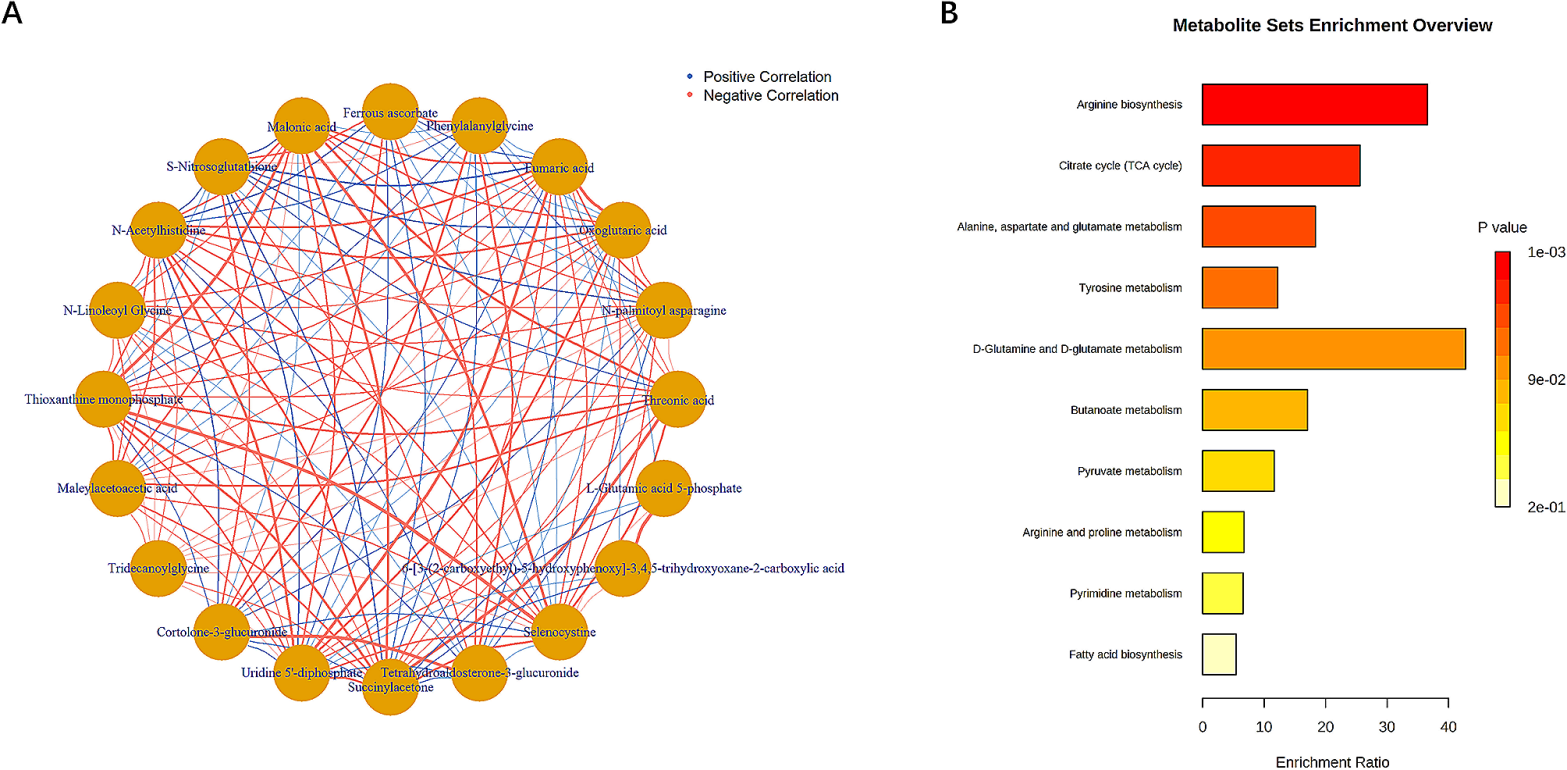
(**A**) Correlation analysis based on metabolic networks, red lines indicate positive correlations and blue lines indicate negative correlations. (**B**) Results of pathway enrichment analysis: colour indicates *p*-value, darker colour indicates smaller *p*-value and stronger significance

To further investigate the interrelationships among significantly different metabolites, we performed correlation analysis using R package ‘corrplot’ and network graph presentation using R package ‘igraph’ (Fig. 4A). The HMDB IDs of the above 20 metabolites were further entered into MetaboAnalyst 5.0, and further metabolic pathway enrichment analysis (Fig. 4B) showed that 20 of the differential metabolites were involved in 10 metabolic pathways, of which 5 metabolic pathways were significantly associated with metabolites (*p* < 0.05): arginine biosynthesis, citrate cycle (tricarboxylic acid [TCA] cycle), alanine, aspartate, and glutamate metabolism, tyrosine metabolism, and D-glutamine and D-glutamate metabolism. The greatest significance was found for the arginine biosynthesis metabolic pathway (*p* = 0.00113).

### Metabolite screening and regression analyses

**Fig. 5:**
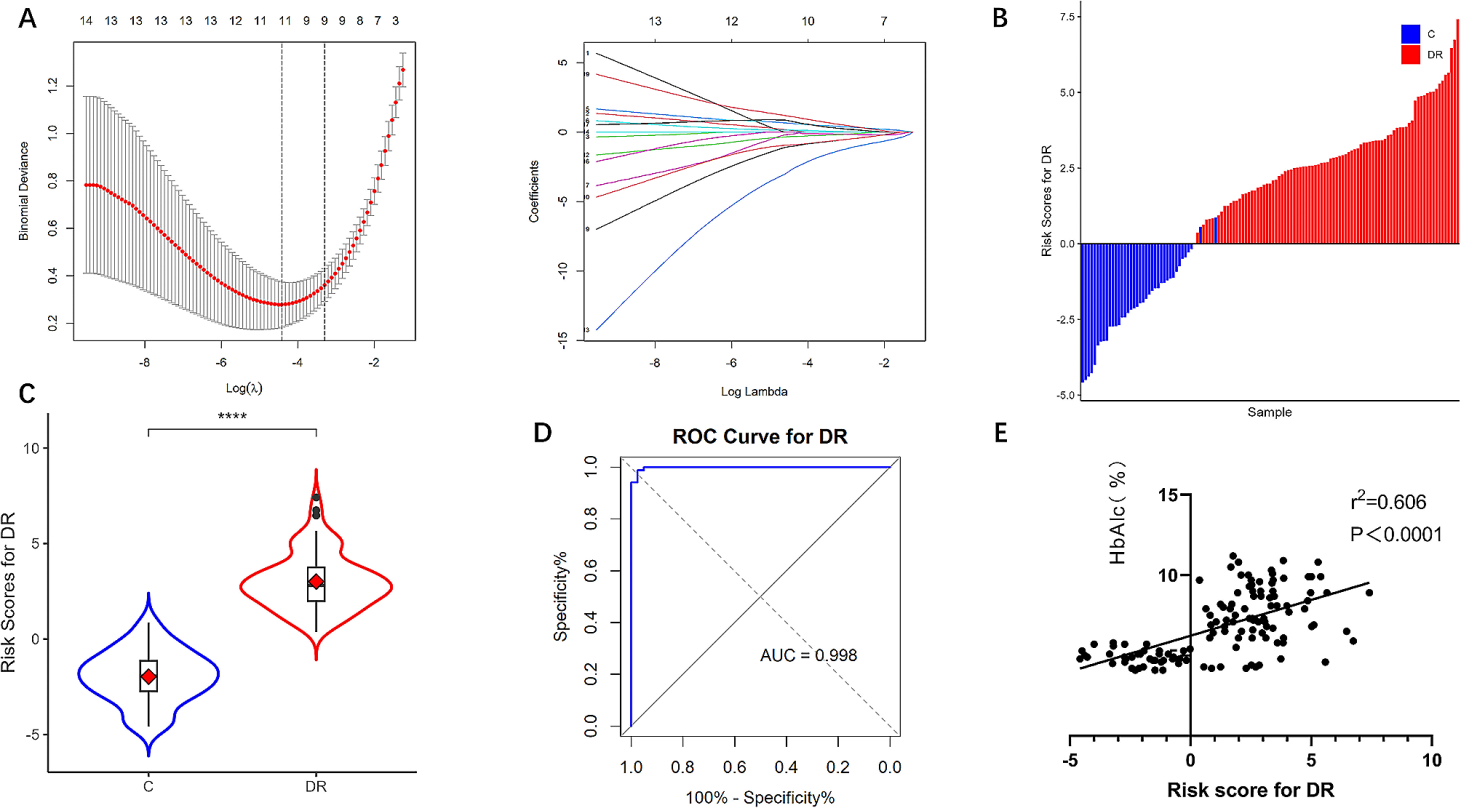
(**A**) Screening of metabolites by LASSO regression. Using the minimum criterion for λ(log) and 1 standard error of the minimum criterion (1-SE criterion), with vertical dashed lines plotted at the optimal values. (**B**) Distribution of risk scores in the DR and control groups. (**C**) Statistical analysis of the distribution of risk scores between the DR and control groups (****p < 0.0001). (**D**) ROC curves were plotted to assess risk score efficacy. (**E**) Linear correlation analysis between risk scores and HbA1c levels. LASSO, Least Absolute Shrinkage and Selection Operator; ROC, receiver operating characteristic

To further elucidate the metabolic characteristics of DR, the LASSO-LR model was used to select diagnostic metabolites and a more refined model was obtained by constructing a penalty function that reduced the regression coefficients of some non-important features to zero. The optimal λ value in the LASSO model was obtained using the minimum error as the criterion. The normalised path of the LASSO-LR model was calculated on a grid of normalisation parameter values, which identified nine differentially expressed metabolites (Fig. 5A). LASSO-LR modelling was performed after taking Ln for the quantitative values of metabolites to obtain the risk score formula for DR: y = 26.50 + 0.43*Ln(phenylalanylglycine) + 0.058*Ln(ferrous ascorbate) -0.62*Ln(N-acetylhistidine) -0.61*Ln(N-linoleoyl glycine) -0.19*Ln(maleylacetoacetic acid) -1.43*Ln(tridecanoylglycine) -0.13*Ln(succinylacetone) + 0.30*Ln(tetrahydroaldosterone-3-glucuronide) + 0.61*Ln(6-[3-(2-carboxyethyl)-5-hydroxyphenoxy]-3,4,5-trihydroxyoxane-2-carboxylic acid). Based on the relationship between the distribution of risk scores and the extent of DR, the incidence of DR was significantly lower in the low-risk score group than the high-risk score group (Fig. 5B), and the risk scores were significantly higher in the DR group than in the control group (Fig. 5C). The ROC curve was plotted to assess the efficacy of the risk score and the AUC was 0.998 (Fig. 5D). Pearson linear correlation analysis (r^2^ = 0.606, *p* < 0.0001) showed that DR risk score was positively correlated with HbA1c levels (Fig. 5E).

**Fig. 6:**
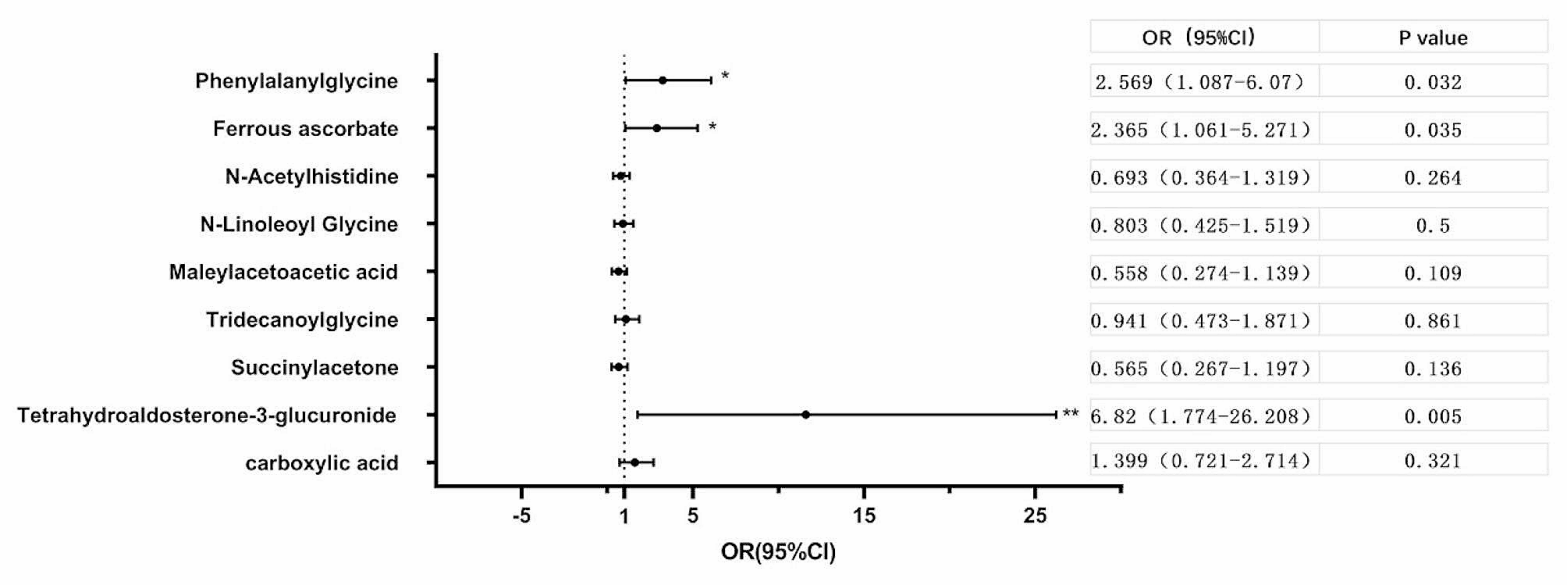
Association between DR vitreous fluid metabolites and systemic metabolic abnormalities. Models were adjusted for age, sex, BMI, insulin therapy, and metformin therapy as cofactors. Error bar: 95% confidence interval. *Indicates *p* < 0.05, **indicates *p* < 0.01. BMI, body mass index; DR, diabetic retinopathy

To further explore the association between vitreous humour metabolites and systemic glycaemic control, HbAlc > 6.5% was used as the outcome variable, and nine metabolite intensities were used as independent variables, respectively, and age, sex, presence of hypertension, BMI, SBP, DBP, TC, TG, HDL-c, LDL-c, RBP, FBG, serum creatinine, and duration of diabetes mellitus as the cofactors adjusted logistic regression model. Phenylalanylglycine, ferrous ascorbate, and tetrahydroaldosterone-3-glucuronide significantly contributed to poorer glycaemic control outcomes (*p* < 0.05) (Fig. 6).

## Discussion

In this study, metabolomic analysis revealed that 20 metabolites, including ferrous ascorbate, phenylalanylglycine, and tetrahydroaldosterone-3-glucuronide, were differentially expressed in DR versus non-DR patients and established a risk score formula based on nine metabolites with the most significant differences, which was useful for diagnosing DR and evaluating DR severity. We further showed that phenylalanylglycine, ferrous ascorbate, and tetrahydroaldosterone-3-glucuronide were risk factors for the level of glycaemic control, highlighting the potential of vitreous fluid biomarkers for disease prediction.

We observed significant differences in arginine biosynthesis; tricarboxylic acid cycle; alanine, aspartate, and glutamate metabolism; tyrosine metabolism; and D-glutamate metabolism pathways in patients with DR compared with controls, suggesting the presence of oxidative stress damage and mitochondrial dysfunction. Previous LC-MS metabolomic studies based on atrial fluid and serum reported similar results [20–22]. Kyoto Encyclopedia of Genes and Genomes results revealed that oxoglutaric acid was involved in arginine biosynthesis; the tricarboxylic acid cycle; alanine, aspartate, and glutamate metabolism; and the D-glutamate metabolism pathway. Fumaric acid is involved in arginine biosynthesis; the tricarboxylic acid cycle; alanine, aspartate, and glutamate metabolism; and the tyrosine metabolism pathway. Maleylacetoacetic acid is involved in tyrosine metabolism. Our results indicate decreased levels of the three substances in the vitreous fluid of patients with DR. Arginine, a semi-essential amino acid, is one of the most glucose-dependent insulinotropic secretagogues and a substrate for nitric oxide synthase and arginase, which can significantly affect vascular endothelial cells through NO production [23]. Elevated proline, ornithine, citrulline, and arginine levels have been found in the vitreous humour in patients with PDR, suggesting that arginine metabolism disturbances are mediators of DR pathogenesis [10]. Glycolysis and tricarboxylic acid cycle are the major pathways of energy metabolism. The α-ketoglutarate (αKG) is a key molecule in the TCA cycle that determines the rate. In the TCA cycle, αKG is decarboxylated to succinyl coenzyme and CO_2_ via αKG dehydrogenase, which is the key control point of the TCA cycle. The αKG can be produced from glutamate via oxidative deamination by glutamate dehydrogenase, where glutamate is a common amino donor [24]. In addition to oxygen partial pressure, αKG, iron ions, and ascorbic acid can modulate the activity of prolyl hydroxylase and hypoxia-inducible factor 1 (HIF-1) expression [25, 26]. Our results revealed low levels of αKG, fumaric acid, and succinylacetone in patients with DR. Furthermore, intermediates in the TCA cycle, including citrate, 2-α-ketoglutarate, L-malate, and succinate, were significantly reduced in patients with diabetes patients in a previous study, suggesting that reductions in the mitochondrial tricarboxylic acid cycle were associated with DR [27]. Alanine may participate in and regulate glucose metabolism through the hypothalamic-pituitary-adrenal axis by stimulating N-methyl-D-aspartate receptors to influence glucose metabolism and subsequently inhibit insulin secretion [28]. Glutamate accumulation in retinal cells contributes to death in many cell types via a variety of mechanisms, producing excess NO and exacerbating oxidative stress [29]. Glutamine is secreted by Müller cells into the extracellular space where it is taken up by neurons and converted into γ-aminobutyric acid (GABA) or glutamate. Hyperglycaemia, the accumulation of glutamate, and reduced glutamine synthetase activity can lead to the loss of neuronal glutamine availability, resulting in glutamate excitotoxicity, causing physiological damage to the retina through oxidative stress, inflammation, and neuronal apoptosis [28].

This is the first report showing that ferrous ascorbate, identified from metabolomic analysis, has an elevated concentration in the vitreous fluid of patients with DR, with an AUC of 0.844 (95% CI = 0.762–0.912) for the diagnosis of DR, and that it significantly contributes to poorer levels of glycaemic control, with implications for DR severity. Several studies have shown that ascorbic acid is involved in free radical scavenging as an antioxidant, and impaired ascorbic acid metabolism in diabetes patients who developed DR causes a downregulation of ascorbic acid levels compared to those who did not develop DR [30, 31]. Except for dioxygen and reactive oxygen species (ROS) products, the only natural mobile electron donor capable of transferring electrons between the plasma and ferritin core is ascorbic acid, and its redox product, the ascorbate radical, and superoxide, as a source of electron donors, are important intermediates in the aerobic release of iron from ferritin by ascorbic acid [32]. Ferrous ascorbate is a strong pro-oxidant that forms the paramagnetic nitrosocorbyl ascorbyl complex Fe-AA-NO with nitric oxide, and the nitrosocorbyl ascorbyl complex may also be an nitric oxide-containing factor involved in vasodilation [33]. Naito et al. [34]. constructed a new model of gastric ulcers by local injection of ferrous and ascorbic acid solutions (Fe/ASA) into the gastric wall to cause ulceration, demonstrating that lipid peroxidation mediated by superoxide radicals generated by the Fe/ASA system played an important role in ulcer development. Hyperglycaemia increases oxidative stress through the overproduction of superoxide in the mitochondrial electron transport chain [35], leading to development of diabetic vascular complications, including DR [36]. The retina itself is very sensitive to oxidative stress due to the constant attack of ROS-producing ultraviolet light and high-energy visible light and the large amount of polyunsaturated fatty acids in the outer segments of the optic rod cells of the retina, which are prone to lipid peroxidation [37]. These events suggest that ferrous ascorbate may play a role in oxidative stress and could be a potential biomarker and new therapeutic target for DR. The role of ferrous ascorbate in the development of DR requires further investigation.

No studies have been conducted to investigate the effect of the potential joint action of the lens, corneal endothelial cells, etc., on the metabolic environment of the DR vitreous fluid. Based on the available literature, the evidence on whether DR and its severity affect corneal endothelial indices is inconsistent [6, 38, 39]. Studies on the correlation between DR severity and corneal endothelial parameters are limited [38]. This discrepancy may be related to the type, severity, and duration of diabetes or the type and severity of DR [40]. Metabolites were affected by the lens and partially originated from circulating plasma before filtration by the ciliary body. There have been no studies related to the metabolic environment of lens metabolites for vitreous fluid. In our study, there was no significant difference in the ocular status of the IOLs in the experimental and control groups, so the lens status did not influence the results in this study. Further experiments are still needed to determine the joint influence of the lens, corneal endothelial cells, and other potential factors on the metabolic environment of the DR vitreous fluid.

In conclusion, we identified 20 differential metabolites through metabolomic analysis of vitreous fluid from 85 patients with DR and 40 patients without diabetes. Subsequently, a model for DR risk assessment was developed based on the nine metabolites with the most significant differences. Our findings suggest that such risk scores, based on molecular profiles, could aid in early disease detection and clinical diagnosis. Notably, ferrous ascorbate, phenylalanylglycine, and tetrahydroaldosterone-3-glucuronide may be potential biomarkers of DR, correlating with poorer glycaemic control. Ferrous ascorbate is a metabolite marker that has not been previously reported to be associated with diabetes mellitus or DR and may be a new target for DR treatment.

### Limitations

The small sample size may have affected the robustness of the study model. These findings should be corroborated by metabolomic analyses with a larger cohort of patients. Furthermore, the sensitivity and specificity of the diagnostic model should be assessed in a larger prospective cohort. As vitreous humour necessitates surgical extraction, only patients undergoing vitrectomy were included. Previous metabolomic analyses used non-diabetes patients as controls, as diabetic patients without DR typically do not require surgery. Consequently, further research with animal models is needed to delineate diabetes’ individual metabolic effects.

## Conclusions

We identified 20 differential metabolites by metabolomic analysis of the vitreous humour of patients with DR and healthy controls and developed a model for DR risk assessment based on the nine most significantly different metabolites. Ferrous ascorbate, phenylalanylglycine, and tetrahydroaldosterone-3-glucuronide are potential biomarkers of DR and are associated with poor glycaemic control. Ferrous ascorbate was identified for the first time as a novel metabolite marker with no previous association with diabetes or DR. Further studies are needed to validate these findings and identify longitudinal associations with the disease.

### Electronic supplementary material

Below is the link to the electronic supplementary material.

Additional File 1

## Data Availability

The datasets used and analysed during the current study are available from the corresponding author on reasonable request.

## Abbreviations

αKG: α-ketoglutarate
BMI: Body mass index
DBP: Diastolic blood pressure
DME: Diabetic macular oedema
DR: Diabetic retinopathy
FBG: Fasting blood glucose
SCR: Serum creatinine
HbAlc: Haemoglobin A1C
HDL-C: High-density lipoprotein
KEGG: Kyoto Encyclopedia of Genes and Genomes
LASSO: Least Absolute Shrinkage and Selection Operator
LDL-C: Low-density lipoprotein
PCA: Principal component analysis
PDR: Proliferative diabetic retinopathy
RBP: Retinol-binding protein
SBP: Systolic blood pressure
ROS: Reactive oxygen species
TCA: Citric acid cycle
TG: Triglycerides
TC: Total cholesterol
