# Supplementary material for: Ferrous ascorbate as a potential biomarker for diabetic retinopathy: a vitreous humour metabolomics study
Source: BMC Ophthalmol. 2024 Jun 24;24:270. doi: 10.1186/s12886-024-03530-6 (PMC11194985; doi:10.1186/s12886-024-03530-6)
Supplement: Supplementary file 1 — Additional File 1 [file 12886_2024_3530_MOESM1_ESM.docx]

**
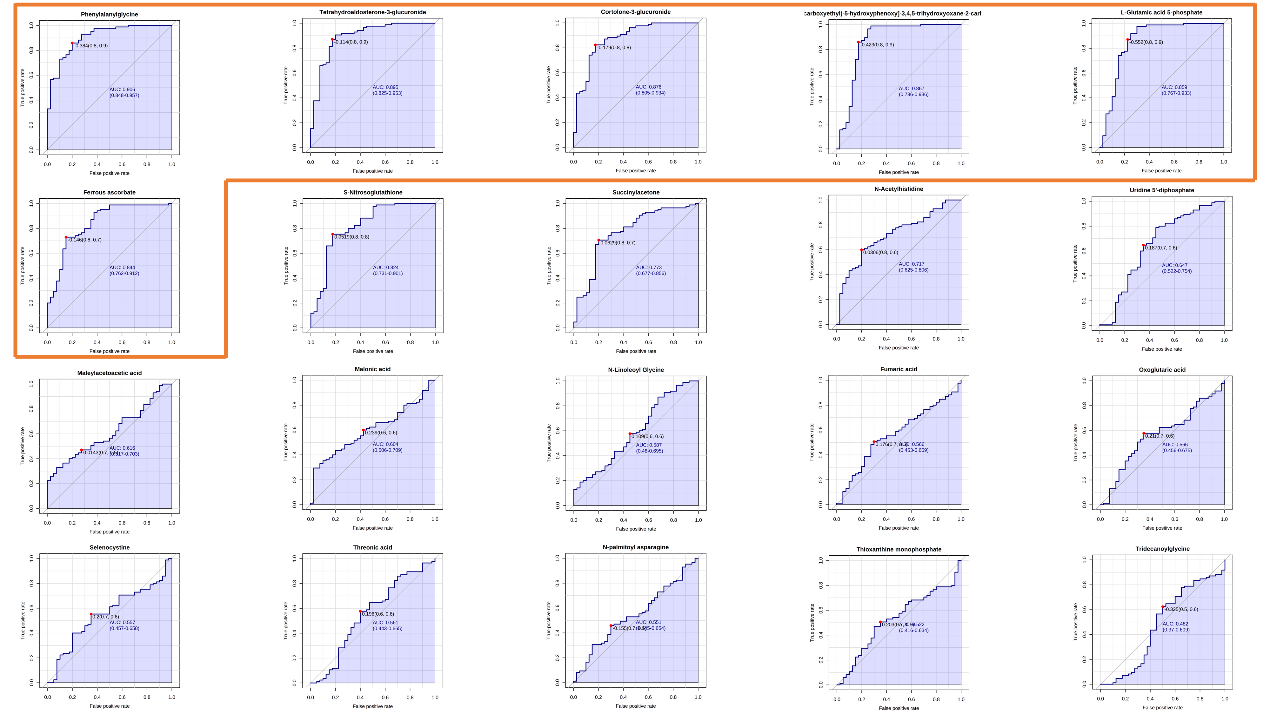
**

**Figure S1.** ROC curves for all differential metabolites. The red boxes indicate the differential metabolites in the top 6 AUC values, which were used as six candidate metabolites for DR diagnostic markers

**Table S1** Liquid phase conditions

| Instrument model | Thermo Scientific Vanquish ultra-high performance liquid chromatograph | |  |
| --- | --- | --- | --- |
| Column | ACQUITY UPLC® HSS T3, 1.8 µm, 2.1×100 mm | |  |
| mobile phase | mobile phase B: ACN  Mobile phase A: H_2_O + 0.1% FA (volume fraction)  Column temperature: 35℃  Flow rate: 0.35 mL/min  Sample volume : 10 µL  Gradient elution as specified in the table below: | |  |
|  |  |  |  |
|  |  |  |  |
| Time (min) | Mobile phase A (％) | Mobile phase B (％) |  |
| 0.0 | 98 | 2 |  |
| 0.5 | 98 | 2 |  |
| 1.5 | 80 | 20 |  |
| 4.0 | 35 | 65 |  |
| 11.0 | 5 | 95 |  |
| 15.0 | 5 | 95 |  |
| 15.1 | 98 | 2 |  |
| 20.0 | 98 | 2 |  |

**Table S2** Mass spectrometry conditions

| Instrument Model | Quadrupole/electrostatic field orbit trap high-resolution mass spectrometer  Q-Exactive |
| --- | --- |
| Ionization mode | ES+/- |
| Capillary temperature (°C) | 320 |
| Spray voltage (V) | 3.7k (+) 3.0(-) |
| Auxiliary gas heater temperature (°C) | 350 |
| Capillary (kV) | 3.0 |
| S-lens RF level | 60 |
| Sheath gas flow rate (Arb) | 35 |
| Auxiliary gas flow rate (Arb) | 10 |

**Table S3** Mass spectrometry acquisition methods

| Ionization mode | Full MS/dd-MS^2^ (Top N) | |
| --- | --- | --- |
| General | | |
| Runtime | 0–20 min | |
| Polarity | Positive/negative | |
| Default charge state | 1 | |
| Full MS | | |
| Resolution | | 70,000 |
| AGC target | | 1e^6^ |
| Maximum IT | | 100 ms |
| Scan range | | 70–1000 m/z |
| dd-MS^2^ | | |
| Resolution | | 17,500 |
| AGC target | | 1e^5^ |
| Maximum IT | | 50 ms |
| Loop count | | 10 |
| TopN | | 10 |
| Isolation window | | 1.5 m/z |
| （N）CE/stepped NCE | | 20, 40, 60 |
| dd Settings | | |
| Minimum AGC target | | 8.00 e^3^ |
| Apex trigger | | 4–8s |
| Exclude isotopes | | on |
| Dynamic exclusion | | 10.0 s |
